# Cognitive Demands of Lower Paleolithic Toolmaking

**DOI:** 10.1371/journal.pone.0121804

**Published:** 2015-04-15

**Authors:** Dietrich Stout, Erin Hecht, Nada Khreisheh, Bruce Bradley, Thierry Chaminade

**Affiliations:** 1 Department of Anthropology, Emory University, Atlanta, Georgia, United States of America; 2 Department of Psychology, Georgia State University, Atlanta, Georgia, United States of America; 3 Department of Archaeology, University of Exeter, Exeter, United Kingdom; 4 Institut de Neurosciences de la Timone, Aix Marseille Université, Marseille, France; Universidade do Algarve, PORTUGAL

## Abstract

Stone tools provide some of the most abundant, continuous, and high resolution evidence of behavioral change over human evolution, but their implications for cognitive evolution have remained unclear. We investigated the neurophysiological demands of stone toolmaking by training modern subjects in known Paleolithic methods (“Oldowan”, “Acheulean”) and collecting structural and functional brain imaging data as they made technical judgments (outcome prediction, strategic appropriateness) about planned actions on partially completed tools. Results show that this task affected neural activity and functional connectivity in dorsal prefrontal cortex, that effect magnitude correlated with the frequency of correct strategic judgments, and that the frequency of correct strategic judgments was predictive of success in Acheulean, but not Oldowan, toolmaking. This corroborates hypothesized cognitive control demands of Acheulean toolmaking, specifically including information monitoring and manipulation functions attributed to the "central executive" of working memory. More broadly, it develops empirical methods for assessing the differential cognitive demands of Paleolithic technologies, and expands the scope of evolutionary hypotheses that can be tested using the available archaeological record.

## Introduction

Enhancement of prefrontal executive control is seen as critical to the emergence of modern human cognition [1–4], but evidence regarding the actual neurophysiological demands of archaeologically-visible behaviors remains scant. Although long tradition [5, 6] links toolmaking to human brain evolution, many recent analyses have concluded that stone tools provide relatively little evidence of pre-modern cognition. For example, it has been argued that Paleolithic technological change is poorly correlated with brain size change [7], that increasing technological sophistication is likely epiphenomenal to underlying changes in social cognition [8], and that technological variation is better explained in terms of economic and environmental factors [9]. Others have concluded that stone tools provide evidence of spatial [10] and procedural learning abilities but not of executive functions [1] or that Paleolithic toolmaking was supported by a specialized domain lacking the “cognitive fluidity” characteristic of modern humans [11]. Still other researchers see evidence of complex cognition in Paleolithic toolmaking, including executive functions associated with prefrontal cortex [4, 12, 13]. Largely missing from this debate is empirical evidence of the cognitive demands of particular stone toolmaking behaviors, leading one recent review to conclude that "links among brain size, cognitive complexity, and technological skill […] are more articles of faith than a hypothesis based on solid middle-range research." [9: 51] To remedy this, we have adopted an experimental neuroscience approach, training modern subjects in Lower Paleolithic stone toolmaking methods and collecting structural and functional brain imaging data as they performed controlled experimental tasks.

Experimental replication of prehistoric behavior is a core research method in archaeology [14, 15] that has been widely used to investigate the techniques [16], skills [17], biomechanics [18], and fracture mechanics [19] involved in the production of flaked stone tools. In order to support inferences about the past, experimental archaeologists aim to identify necessary relations between behavioral variation and material traces of the kind that can be observed in the archaeological record. The application of neuroscience methods to experimental archaeology allows more detailed characterization of this behavioral variation, including physiological [20–22] and structural [23] responses in the brain, and thus expands the range of inferences that can be drawn from archaeological evidence. Here we seek to identify brain systems supporting particular aspects of stone toolmaking competence, and to relate variation in the functional response of these systems to variation in the experimental artifacts produced.

Our previous research examined brain responses to naturalistic stone toolmaking behavior execution [20, 21] and observation [22], identifying a bilateral frontoparietal network supporting stone toolmaking and documenting increased response to more recent stone technology. These findings support an evolutionary scenario in which perceptual-motor adaptations enabled the initial stages of human technological evolution whereas later developments were dependent on enhanced cognitive control [24], and particularly the inhibitory and task-set shifting functions of the right inferior frontal gyrus. Research to date has not, however, indicated the involvement of dorsolateral prefrontal cortex regions thought to support executive functions such as relational and temporal abstraction [25], or information selection, monitoring and updating [26], that are attributed to the "central executive" of working memory [1].

Prior experiments prioritized ecological validity and studied naturalistic tasks to reveal generalized demands over relatively extended timescales (20s ~ 40m), but were not designed to dissect task sub-components or detect infrequent but potentially important brain responses (e.g. those associated with a small number of critical strategic choices). To better focus on these questions here, we adapted a behavioral paradigm developed by Bril and colleagues [17] for use as an fMRI experiment. Subjects were shown predictions of toolmaking action outcomes and asked to make judgments about them. By varying questions we manipulated cognitive task demands across identical stimuli, distinguishing between judgments on the *physical accuracy* of predicted outcomes vs. their *strategic appropriateness* in achieving toolmaking goals. This manipulation approximates a conventional archaeological distinction between *savoir-faire* (know-how) and *connaissance* (knowledge about) in stone toolmaking [27], which is itself loosely convergent with contrasts of procedural vs. declarative memory and perceptual-motor vs. cognitive skill [28] developed in other disciplines. We anticipated that the strategy task especially would rely on the selection, monitoring and updating of abstract technological concepts, and thus elicit greater prefrontal response, whereas the prediction accuracy task would rely on internal simulation and thus elicit greater perceptual-motor response.

Brain responses to the observation of skilled actions are modulated by experience [22, 29], and accounts of expert cognition based on the formation of task-specific knowledge structures ("chunking" [30]) suggest greater working memory demands during learning vs. expert performance. To address this, we employed a longitudinal design, training subjects for two years in a variety of archaeologically attested Paleolithic toolmaking methods and conducting fMRI experiments at the start (T1), mid-point (T2), and end (T3) of training. This demanding training program limited sample size but enabled investigation of the acquisition of a real-world, evolutionarily-relevant skill in a manner not previously achieved in either archaeology or neuroscience. We evaluated behavioral and brain responses to stimuli representing simple flake production (cf. “Oldowan”, “Mode 1”, “Mode C” [31], hereafter "Oldowan") and refined biface shaping (cf. “Later Acheulean handaxe”, “Mode 2”, “Mode E2” [31], hereafter "Acheulean"). We predicted an interaction between Task (Prediction vs. Strategy), Technology (Oldowan vs. Acheulean), and Time (T 1, 2, 3) such that prefrontal response would be greater for the Strategy task, especially with respect to the more complex Acheulean technology and at earlier stages of skill acquisition. Our training program also allowed us to study tools produced by our research subjects outside the scanner [32]. We expected that individual performance on our MRI tasks would be predictive of actual success with stone toolmaking.

## Methods

### Subjects and training

Subjects were recruited from undergraduate and postgraduate programs in Archaeology at Exeter University. Subjects were ages 18–25 at the time the first scan was collected, 5 male and 1 female. All were right-handed by self-report and subsequent observation, had no neurological or psychiatric illness, and provided written informed consent before the study and the study was approved by the Ethics Committee at Exeter University. Imaging took place at the Wellcome Department of Imaging Neuroscience in London. All subjects provided additional written informed consent for imaging data collection and the research was approved by the National Hospital for Neurology and Neurosurgery and Institute of Neurology Joint Research Ethics Committee (Reference #: 1825/003).

**Fig 1:**
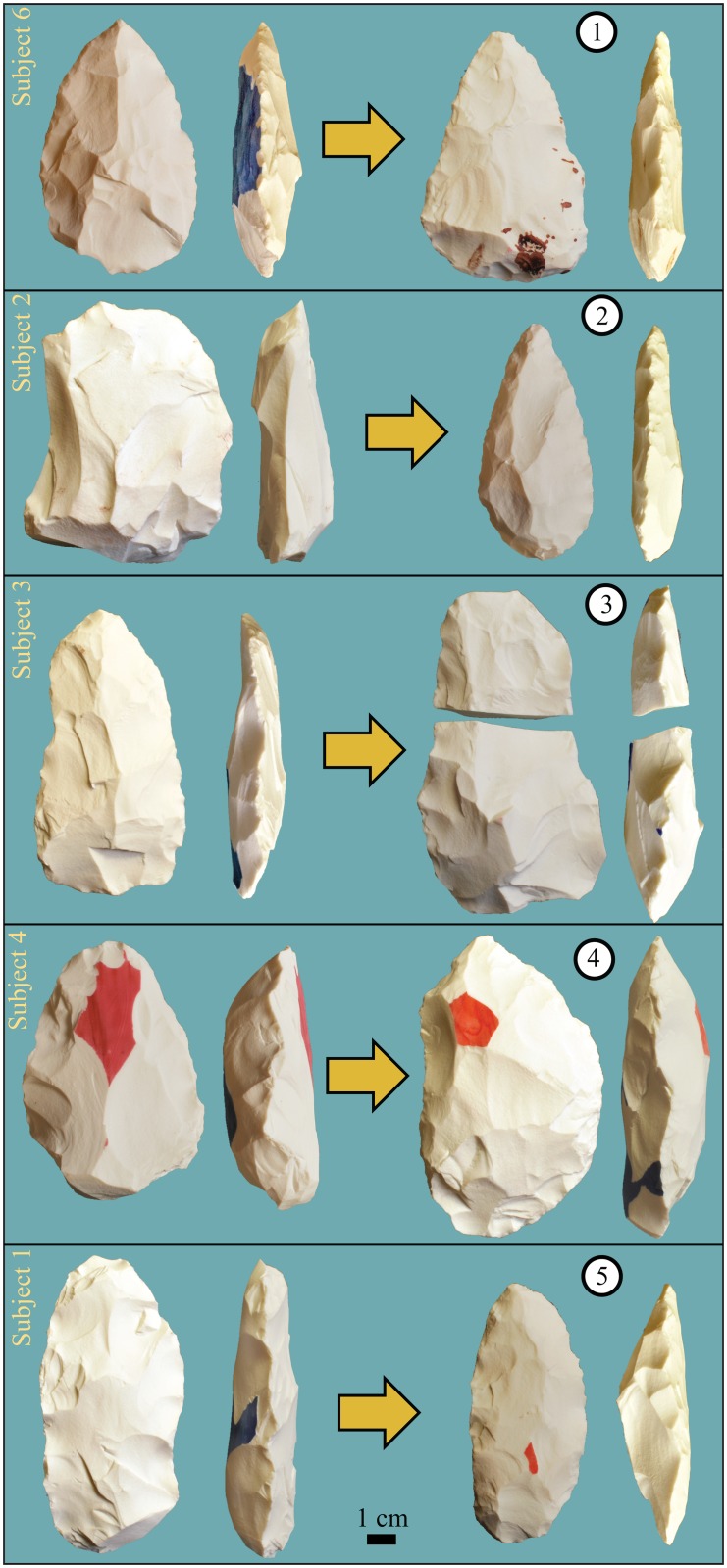
Handaxes produced for the first (left) and last (right) evaluations, ranked by T3 fMRI task performance (circled numbers).

Stone toolmaking involves striking a stone “core” with a “percussor” of bone, antler, or stone to detach controlled flakes and incrementally achieve design goals. Training was conducted by BB and NK, as detailed in [32], and included instruction, coaching, and demonstration as well as independent practice, which was recorded by subjects in a log book. Pedagogical techniques were not restricted in any way and the explicit aim of instruction was to elicit maximum skill development by drawing on the extensive tool-making and training experience of the instructors. Toolmaking methods introduced to the subjects included: 1) basic flake production, comparable to the earliest known (Oldowan) tools of *Homo habilis* 2.6–1.5 million years ago (mya); 2) “Handaxe” making, comparable to the Acheulean tools of *Homo erectus* and *Homo heidelbergensis* 1.7–0.25 mya; and 3) “prepared core” flake production, comparable to the Levallois tools of Neanderthals and early *Homo sapiens* <0.25 mya. Training was naturalistic and self-paced, leading to intersubject variation in the duration and content of practice. Learning was assessed through comparison of artifacts produced (Fig. 1) during formal evaluations before and after training in each technology [32]. For Oldowan evaluations, subjects were asked to detach five flakes from a flint core. For Acheulean and Levallois evaluations, subjects were asked to produce a tool (handaxe or preferential Levallois flake) from a standardized porcelain core [33].

Paleolithic toolmaking occurred over a vast time period and many millions of square miles, and encompasses substantial variation that could not be included in our training program. The methods we did select are considered broadly representative of Lower and Middle Paleolithic technology, and details of the production techniques employed closely match those documented in specific archaeological collections [34]. We thus consider our training protocol to be both generally representative and specifically accurate in re-creating learning challenges actually faced by Paleolithic toolmakers.

### Experimental design

Stimuli were 1.5-second videos of rotating stone cores (Oldowan, Acheulean or Levallois) marked with coloured cues indicating the next strike of a notional toolmaker: a red dot indicated the intended point of impact, and a white area showed the flake predicted to result from percussion at this point [17]. Levallois stimuli were omitted from subsequent analyses, because instructor evaluations indicated that subjects failed to develop basic proficiency in this technology [32]. Before each scan (T1, 2, 3) subjects received a standard briefing on the technologies and experimental tasks (Prediction: "if the core were struck in the place indicated, is what you see a correct prediction of the flake that would result?"; Strategy: "is the indicated place to hit the core a correct one given the objective of the technology?"). In the scanner, stimuli were presented in blocks of 4. For each block subjects were given a 2 s text prompt indicating which Technology and Task they would be responding to, followed 0.5 s later by a series of 4 stimulus presentations (0.25 s black screen, 5 s video, 0.25 s black screen, 2.5 s response screen). Response screens indicated which button (left/right) to use for yes and no, in a randomized fashion.

### MRI data acquisition

Each scanning time included seven acquisitions: a fieldmap (double echo FLASH), four functional runs (EPI, FOV 192×192 mm^2^, inplane voxel size 3×3 mm^2^, 48.0 3-mm tick descending axial slices without gap, TR 3264.0 ms, 136 repetitions) covering the whole brain, a T1 anatomy (MPRAGE) and a Diffusion Tensor Imaging scan [23].

### fMRI data analysis

SPM8 and associated toolboxes were used for the analysis of MRI data [35]. Realignment and unwarping procedures were applied to fMRI time series to correct for both the static distortions of the magnetic distortions with the voxel displacement map obtained from the fieldmap and the movement-induced distortions of the time series [36]. The high-resolution anatomical images were coregistered with the mean EPI image, before being segmented using VBM8 toolbox. For each subject, the three anatomical images were realigned and a mean image created. The DARTEL toolbox was used for diffeomorphic registration of the six mean anatomical images. Realignment parameters, DARTEL transformations from original to template image and normalization parameters of the DARTEL template were combined for the normalization of functional time series [37] with a 8-mm FWHM Gaussian kernel smoothing and voxel resampling to 1.5 mm^3^. A mean anatomical volume image was created by averaging the individual anatomies transformed and normalized according to DARTEL parameters.

For each Subject and Time analysis a separate analysis was run (first-level analysis), in which the six experimental conditions (2 Tasks “Prediction” & “Strategy” by 3 Technologies “Oldowan” by “Acheulean” by “Levallois”) were modeled as 32-second boxcar functions. Condition regressors were convolved with the canonical hemodynamic response function with a high pass filter (128 s). Contrast images between conditions and rest for each of the four recording sessions per subject and time were used in second-level repeated-measure analysis of variance using GLMFlex toolbox, with Time (T1, 2, 3), Task and Technology as factors of interest and Sessions and Subjects as random factors.

The *conn* toolbox [38] was used to investigate the functional connectivity of the left superior frontal gyrus cluster. Sources of confounding variance (estimated motion parameters; BOLD signals in grey matter, white matter and cerebrospinal fluid resulting from the VBM segmentation; main effects of the tasks) were removed from the smoothed time series through linear regression. Data were high-pass filtered (cut-off 128 s) to eliminate low frequency drifts. Mean time courses were extracted in the region of interest and correlated with activity in all voxels creating whole-brain maps of regression coefficients for each Subject, Time, Task and Technology. Regression coefficient images were used in a repeated-measure analysis of variance using GLMFlex toolbox, with Time, Task and Technology as factors of interest and Subjects as a random factor.

### Probabilistic tractography

A binarized mask of the left superior frontal gyrus cluster was used as a seed for probabilistic tractography using FSL [39], a software library of analysis tools for neuroimaging data. Each subject’s B0 image was registered to their T1-weighted structural image using a 6-degree of freedom, rigid-body registration computed by FSL’s FLIRT algorithm. T1 images were first registered to the MNI 1mm template using a 12-degree of freedom, affine registration computed by FLIRT, which was then used to constrain a nonlinear warp computed by FSL’s FNIRT algorithm. The T1-to-MNI and B0-to-T1 registrations were then inverted and concatenated to warp MNI-space functional activations into individual subjects’ diffusion space. These diffusion-space activation masks were used to seed probabilistic tractography analyses using probtrackx, a tool in FSL’s FDT software package. Tracts were thresholded at 0.1% of the waytotal, binarized, warped into MNI template space, and summed. We measured, in native diffusion space, the number of above-threshold voxels from each tract that reached each of the gray matter regions included in the AAL atlas [40].

## Results and Discussion

### Behavior

**Fig 2:**
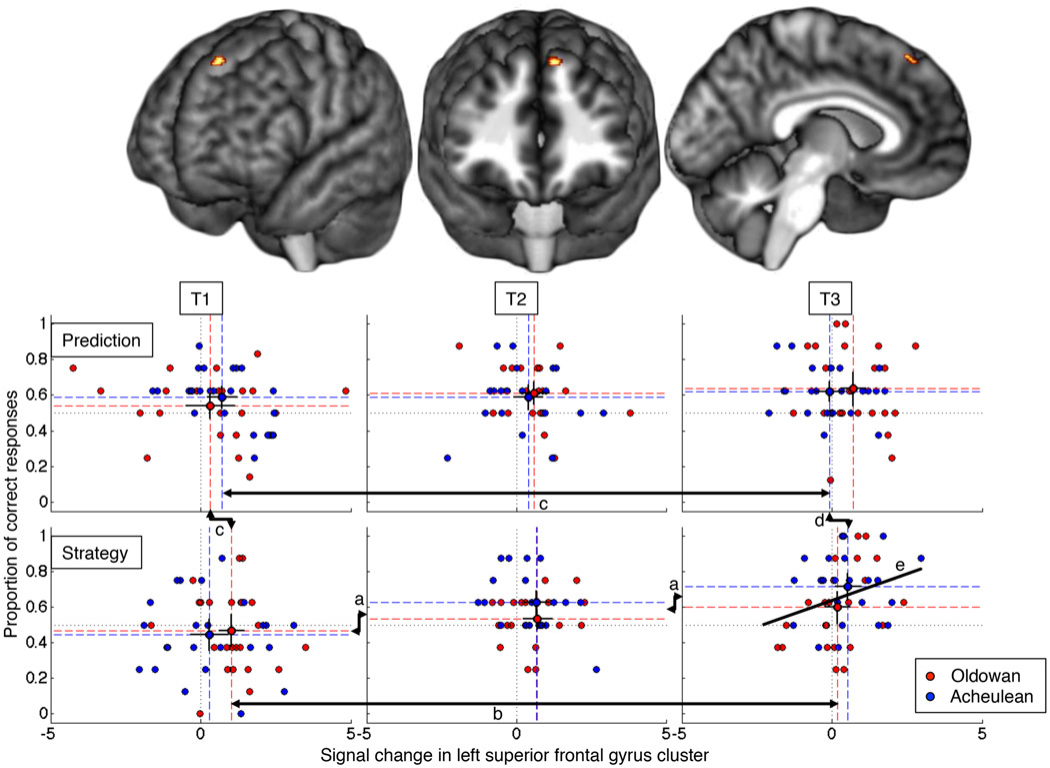
Location of the significant 3-way interaction in left SFG (top) and the relation of fMRI signal change to task performance (bottom). Arrows *a—d* indicate significant pairwise differences across time and tasks. *e* is a regression line (r = 0.294).

The ratio of correct over expressed responses was calculated for each Subject, Time, Task, Technology and Session. Within subjects mixed effect analysis of variance, using Session as a random variable, revealed a significant effect of Time (F(2,257) = 12.3, *p* <. 001) and an interaction between Time and Task (F(2,257) = 3.3, *p* <. 04) on the proportion of correct responses (Fig. 2). The three way interaction Time by Task by Technology did not reach significance (F(2,257) = 2.5, *p* = .085). Other effects and interactions were also non-significant (*p* > 0.05). Post-hoc pairwise comparisons showed that the proportion of correct responses for the *Strategy* Task was significantly lower than for physical *Prediction*. All pairwise comparisons between Times were significant for the *Strategy* Task (Fig. 2a), but none for the *Prediction* Task. Thus, the judgments of strategic appropriateness were more difficult at the outset, but improved with training whereas judgments on the physical accuracy of predictions did not.

To assess the real-world relevance of our experimental tasks, we compared task performance with subjects’ actual tool production. For Oldowan flaking, we measured total area (Length x Breadth) of flakes produced. For Acheulean handaxe-making, we measured the width/thickness ratio ("refinement") of finished artifacts, a conventional index of skill for in bifacial tool production [34]. There was a clear increase in Oldowan flake area from the first to last evaluation (mean = 6253 mm^2^ vs. 19008 mm^2^, each subject increased flake area by at least 1.9x). Handaxe refinement showed no such trend (mean = 2.23 vs. 2.25), although more qualitative progress by individuals seems apparent (Fig. 1). Success at Oldowan flake production was not significantly correlated with performance rank on Oldowan stimuli during associated fMRI scans (Spearman’s rho = 0.436, df = 11, p = 0.09, one-tailed), especially when Strategy (Spearman’s rho = 0.360, df = 11, p = 0.138, one-tailed) and Prediction (Spearman’s rho = -0.146, df = 11, p = 0.334, one tailed) tasks are considered separately. In contrast, subjects who produced relatively thinner handaxes scored better on Acheulean stimuli in the scanner (Spearman’s rho = 0.750, df = 9, p = 0.010, one-tailed) including both Strategy (Spearman’s rho = 0.619, df = 9, p = 0.038, one-tailed) and Prediction (Spearman’s rho = 0.583, df = 9, p = 0.050, one tailed) tasks. Thus, our training group gained practical competence in Oldowan flaking irrespective of ability to correctly judge flake predictions or strategy. Conversely, there was no measurable group-level increase in handaxe making skill, but individual success was linked with the ability to make technological judgments as measured in our paradigm. Detailed data on all artifacts produced during training are presented in [32].

### fMRI response and relation to behavior

Factorial analyses identified significant main effects of Time and Technology as well a three-way interaction between Time, Task and Technology (*p* < 0.001 uncorrected, extent > 75 mm^3^) as predicted by our hypothesis. Effects of Time and Technology were observed in occipital, parietal and premotor cortex and are consistent with previous work on perceptual learning generally and stone toolmaking specifically.

#### fMRI main effect of Technology

The main effect of technology was associated with clusters in the occipital and parietal cortex. However, parietal effects were small, and failed to reach significance in post-hoc comparisons. Occipital effects were localized in the left (x,y,z = -27, -91, 25; z-score = 3.76; extent = 316 mm^3^) and right (x,y,z = 36, -90, 18; z-score = 3.57; extent = 29 mm^3^) middle occipital gyrus (MOG, BA 19). This portion of dorsal middle occipital gyrus comprises early visual association cortex, and it is likely these activations reflect low-level differences in the visual properties (e.g. size and shape) of Oldowan vs. Acheulean stimuli. Right MOG activity was significantly correlated with individual performance on tasks involving Acheulean stimuli (n = 136, Pearson's r = 0.322, p < 0.001), possibly indicating that attentional modulation played some role in variation of response across subjects. Other Region by Technology correlations with performance were not significant.

#### fMRI main effect of Time

**Fig 3:**
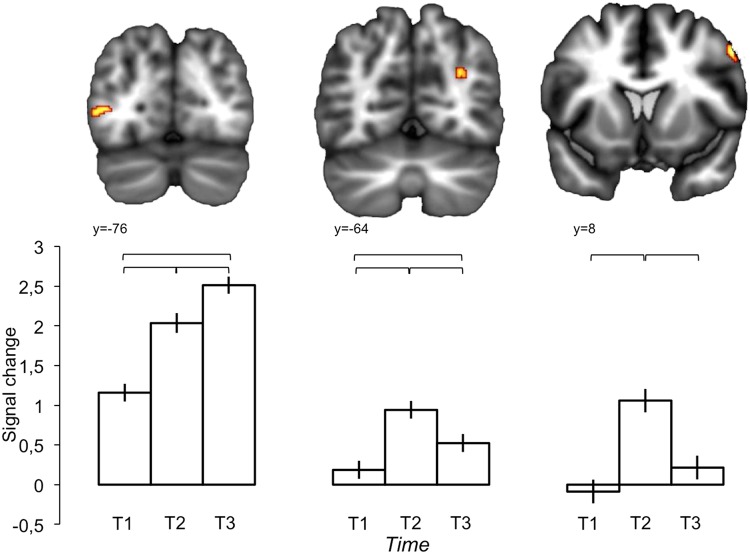
fMRI main effect of Time in (from left to right) left middle occipital gyrus, right posterior intrapareital sulcus, and right precentral gyrus. Brackets indicate significant post-hoc comparisons.

Fig. 3 depicts brain regions showing a main effect of the Time of scanning. Two patterns are evident. First, activity in the left ventral MOG (BA 19) showed a significant increase across each time-step. Attention modulates activity in visual cortex [41], and it is likely that these increases reflect response enhancement arising from training-related changes in visual attention to stimulus features. This would be consistent with numerous studies indicating changes in neural activity associated with perceptual learning [42]. A previous FDG-PET study of stone toolmaking skill acquisition [20] found similar training-related activity increases in MOG and reached similar conclusions. Consistent with this interpretation, left MOG response was weakly, but significantly, correlated with behavioral performance (n = 272, Pearson's r = 0.140, p = 0.021). This overall correlation was specifically driven by a relationship with the Strategy task (n = 136, Pearson’s r = 0.287, p = 0.001), whereas there was no correlation with Prediction (n = 136, Pearson’s r = -0.034, p = 0.693) considered separately. Thus it appears similar perceptual strategies were deployed across tasks, but were only effective for Strategy.

Second, activity at the fundus of the posterior part of the right posterior intraparietal sulcus (attributable to functional area IPS0) and in the right precentral gyrus (PrCG) showed initial increases from T1 to T2, followed by reduction from T2 to T3. This parallels structural changes observed in the same subjects using DTI [23], which showed T1 to T2 increases followed by symmetrical T2 to T3 decreases in white matter fractional anisotropy within branches of the superior longitudinal fasciculus leading into precentral gyrus and posterior parietal cortex. These anatomical changes correlated with subjects’ hours of practice prior to each scan (training was most intense before T2) and appear to reflect transient responses to the perceptual-motor demands of stone toolmaking practice. It is likely that the observed effect of Time on frontoparietal activity here reflects a corresponding functional response.

Frontoparietal activity, including PrCG, supramarginal gyrus (SMG) and intraparietal sulcus (IPS), has been a consistent result in FDG-PET [20, 21] and fMRI [22] studies of stone toolmaking. We have previously attributed such activations to demands for grasp control (PrCG), visuospatial processing (posterior IPS), and sensorimotor transformation (SMG) in the coordinated control of action. In our current paradigm, which involved judgments about visually presented tools without actual prehension, PrCG response was modulated by experience but did not correlate with performance. This is consistent with the well-documented participation of premotor cortex in the perception of graspable objects [43], and its modulation by experience with object function [44]. Activity in right IPS0, a retinotopic visual area modulated by spatial attention [45] and preferentially responsive to tools [46], did correlate with behavioral performance (n = 272, Pearson's r = 0.143, p = 0.018). As with left MOG, this relationship was driven by correlation with the Strategy task (n = 136, Pearson’s r = 0.246, p = 0.004), and not Prediction (n = 136, Pearson’s r = 0.006, p = 0.947). It is unclear why visual areas in right IPS0 and left MOG display different patterns of response to training, although similar variability across studies of perceptual learning is thought to reflect the existence of multiple learning stages [47] with different effects at different locations along visual pathways [48]. It is also notable that IPS0 and PrCG activations are both in the right hemisphere. This is a consistent feature of frontoparietal activations associated with stone toolmaking [21, 24], and stands in contrast to the left lateralization of everyday tool-use [49].

#### fMRI Interaction of Time, Task and Technology

Consistent with our research hypothesis, we observed a three-way interaction in the left superior frontal gyrus (lSFG: x, y, z = -8, 38, 49; z-score = 3.36; extent 22 voxels), a prefrontal region implicated in cognitive control functions [50] including working memory [51] (Fig. 2, top). Post-hoc comparisons show that response to Oldowan Strategy (OS) was initially high and decreased through time (Fig. 2b), whereas response to Acheulean Strategy (AS) was initially lower and remained constant. At the same time, response to Acheulean Prediction (AP) decreased through time (Fig. 2c), but Oldowan Prediction (OP) did not. As a result, OS was greater than OP at T1 (Fig. 2c) whereas AS was greater than AP at T3 (Fig. 2d). This complex pattern likely reflects experimentation with different cognitive strategies over learning, as is typical of early/intermediate stage skill acquisition [22]. In fact, the relationship of lSFG activity to actual task success suggests (Fig. 2) that much of this experimentation was ineffective and/or misguided. SFG activity was uncorrelated with Prediction success at any time point, consistent with the expectation that this task should require perceptual-motor simulation rather than cognitive control. Conversely, lSFG activity was positively correlated with Strategy success (Fig. 2e), consistent with the expectation that this task demands the cognitive manipulation of information, but only at T3 (n = 48, Pearson's r = 0.294, p = 0.042) when (some) relevant concepts had been learned and performance was at its highest.

### Probabilistic tractography and functional connectivity

Intrinsic functional connectivity analyses have identified large-scale functional networks in the human brain [52], including a control network associated with planning and cognitive control, a dorsal attention network associated with external attention, a default network associated with internal attention (e.g. memory and prospection), and a somatomotor network involved in motor control. It has been proposed that lSFG is a key region supporting interaction between default and control networks during goal-directed cognition [50, 53], which would be consistent with its involvement in our tasks. We thus predicted that the lSFG cluster identified in our fMRI analysis would: 1) be anatomically connected with default and control networks, and 2) show shifts in functional connectivity with these networks during task performance.

**Fig 4:**
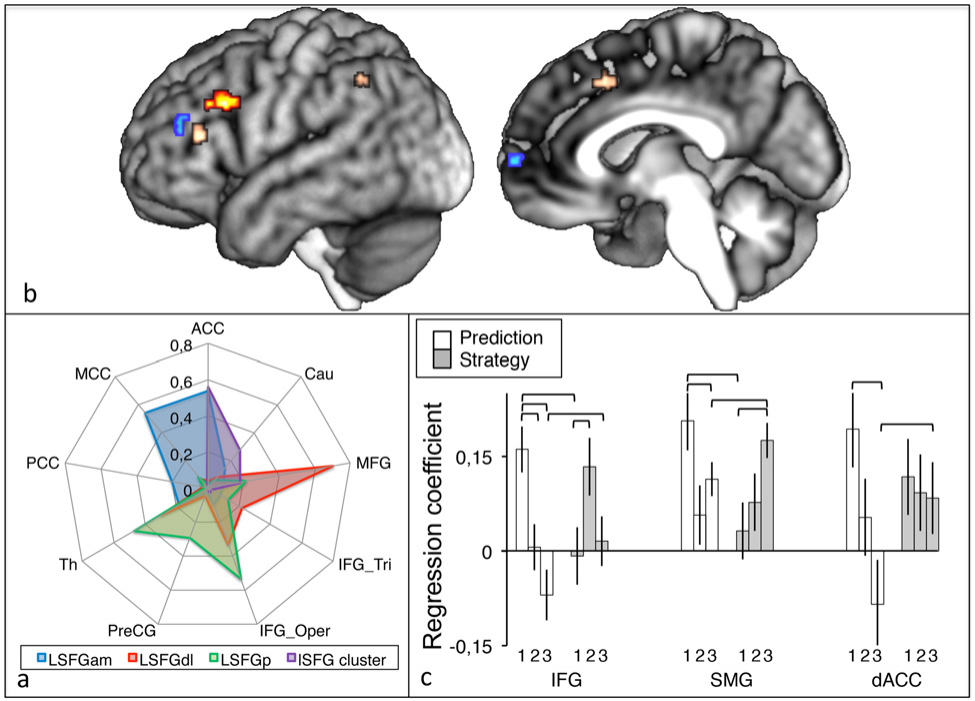
Anatomical and functional connectivity of the left SFG cluster. (a) Radar plot of top targets for SFG sub-regions using data reported by Li et al. [40] (blue: lSFGam, red: lSFGdl, green: lSFGp) and our own analysis of the lSFG cluster reported here (purple). ACC: anterior cingulate cortex, Cau: caudate, MFG: middle frontal gyrus, IFG_Tri: inferior frontal gyrus *pars triangularis*, IFG_Oper: inferior frontal gyrus *pars opercularis*, PreCG: precnetral gyrus, Th: thalamus, PCC: posterior cingulate cortex, MCC: middle cingulate cortex. (b) Surface renders of significant experimental effects on functional connectivity (hot scale: increased for Prediction vs. Strategy, cold scale: increased for Strategy vs. Prediction, tangerine: Task x Time interaction). (c) Regression coefficients for the Task x Time interaction with significant post-hoc comparisons indicated by brackets.

To investigate connectional anatomy, we used our lSFG cluster as a seed for probabilistic tractography. The top three cortical targets identified for our cluster were elements of the control (anterior cingulate cortex, middle frontal gyrus) and default (inferior frontal gyrus) networks, supporting the hypothesis that this portion of lSFG enables coupling of control and default networks. Li et al. [54] parcellated SFG into three sub-regions: anteromedial (SFGam; connected with anterior and mid-cingulate cortices assigned to control and the default networks), dorsolateral (SFGdl; connected with middle and inferior frontal gyri linked to the control and default networks), and posterior (SFGp; connected with the precentral gyrus and frontal operculum of the somatomotor network). We compared the connectional fingerprint of our cluster with reported values for these three sub-divisions and found it to be intermediate between SFGam and SFGdl (Fig. 4a), consistent with its intermediate location on the sub-region probability map.

To investigate functional connectivity, we conducted a factorial analysis of maps of regression coefficients with lSFG activity during stimulation blocks. We observed significant (*p*<0.001 uncorrected, extent > 75 mm^3^) effects of Task in frontal cortex and interactions between Time and Task in frontoparietal cortex (Fig. 4b) as well as interactions between Time and Technology in the middle temporal gyrus and cerebellum, and a complex three-way interaction between Time, Task, and Technology in right SFG. The observed effect of Task and its interaction with Time are perhaps the most relevant results for the current investigation, since these factors were significant sources of behavioral variation and influenced its correlation with lSFG activity (see above).

#### fcMRI Main Effect of Task

Task effects on functional connectivity were seen in regions attributed [52] to default (medial frontopolar cortex) and control networks (posterior and anterior middle frontal gyrus). Increased functional connectivity during Prediction vs. Strategy tasks (Fig. 4b, hot color scale) was seen in the left posterior middle frontal gyrus (dorsal anterior premotor cortex, BA 8: x, y, z = -40, 16, 33; z-score = 5.56; extent 41 voxels) whereas increased connectivity for Strategy vs. Prediction (Fig. 4b, cold color scale) was observed in left anterior middle frontal gyrus (mid-DLPFC, BA 9/46: x, y, z = -49, 40, 21; z-score = 4.09; extent 25 voxels) and medial frontopolar cortex (mFPC, BA 10: x, y, z = 8, 60, 4; z-score = 3.92; extent 25 voxels). Insofar as frontal cortex function is organized along a posterior-to-anterior gradient of increasing cognitive abstraction [25], this pattern is consistent with the expectation of greater demands for abstract information processing in Strategy vs. Prediction tasks.

#### fcMRI Interaction of Task and Time

Interactions between Task and Time (Fig. 4b, tangerine color) were observed in left inferior frontal gyrus (IFG, BA 45: x, y, z = -48, 30, 26; z-score = 3.23; extent 24 voxels), posterior supramarginal gyrus (BA 40: x, y, z = -40, -50, 44; z-score = 2.96; extent 22 voxels), and the posterior portion of dorsal anterior cingulate cortex (dACC, BA 24: x, y, z = -6, 18, 43; z-score = 3.11; extent 25 voxels), brain regions involved in executive planning [55] and monitoring [56] functions, including tool-use planning [49], working memory, and language [57] tasks. Post-hoc comparisons (Fig. 4c) show that, for Prediction, functional connectivity with each of these regions was initially high but decreased with Time whereas for Strategy it was initially low but held steady or increased. As a result, T3 connectivity is greater for Strategy vs. Prediction in each region. The decreases in functional connectivity across time for Prediction are not paralleled by significant change in behavior suggesting a shift between equally viable cognitive strategies. Conversely, neutral to positive changes in functional connectivity for the Strategy task were accompanied by incremental increases in behavioral performance, suggesting a more uniform role across time.

#### fcMRI Interaction of Time and Technology

**Fig 5:**
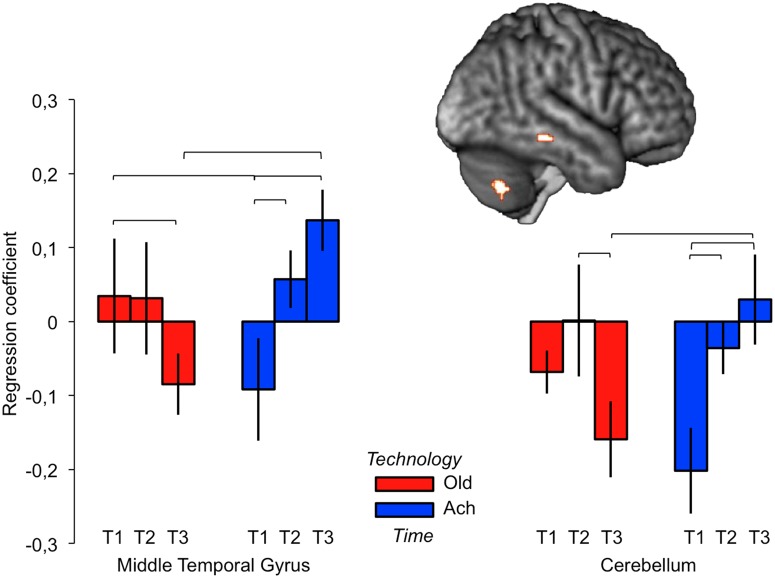
fcMRI interaction of Time and Technology in middle temporal gyrus and cerebellum. Brackets indicate significant post-hoc comparisons.

Fig. 5 depicts brain regions for which functional connectivity was affected by the interaction of time and technology, specifically the right middle temporal gyrus (MTG) and lateral cerebellar cortex. The MTG is important in the representation of conceptual knowledge, including the association between objects (especially tools [58]) and related actions [59]. Increased of functional connectivity between lSFG and MTG over time is seen for Acheulean stimuli only and irrespective of task. We speculate that this could represent increasing reference to semantic knowledge about handaxe making that was acquired over training. We also note that the MTG cluster reported here approximates one node in the cortical default network [60] and that potions of lateral cerebellum may also be linked to this network [61]. Again speculatively, this might suggest an increased role for introspective access to semantic knowledge following training.

#### fcMRI Interaction of Time, Task and Technology

**Fig 6:**
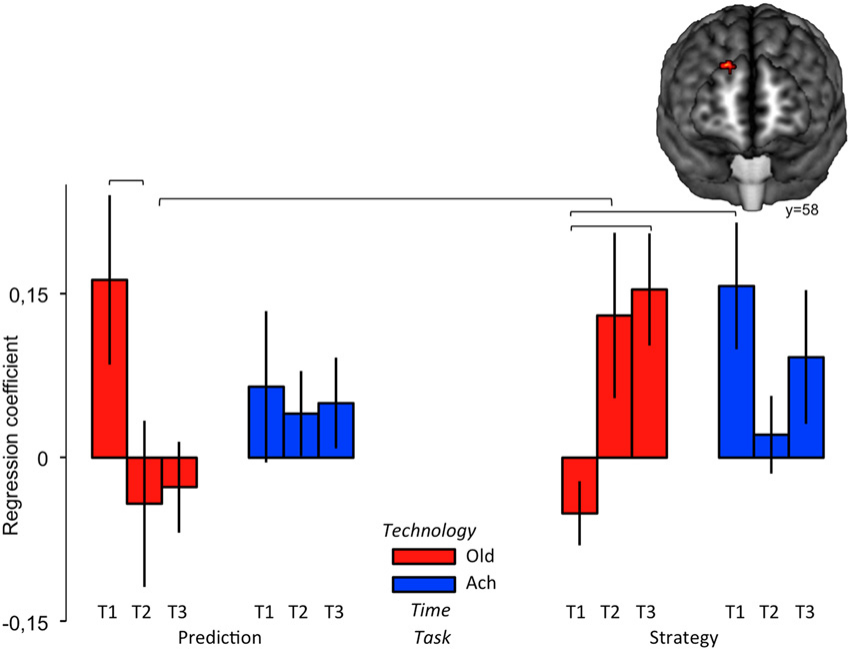
fcMRI interaction of Time, Technology, and Task in right Superior Frontal Gyrus. Brackets indicate significant post-hoc comparisons.

Fig. 6 shows the portion of right superior frontal gyrus (rSFG) where a significant three way (Time, Task, Technology) interaction effect on functional connectivity with lSFG was observed. This cluster is likely located in the right homolog of the functional cortical area containing the lSFG seed. Substantial functional connectivity via callosal connections between the regions is thus expected, and is consistent with the bilateral/bimanual coordination required for successful stone toolmaking [62]. However, the complex patterning of this connectivity across conditions is unexpected. One possible explanation for this unexpected complexity could be that the functional importance of interhemispheric coordination varied over the course of learning as subjects experimented with different behavioral strategies. For example, recent lesion work has linked rSFG to the self-focused reappraisal of negative emotions, perhaps reflecting a more general cognitive role in inhibition [63]. We have previously argued that inhibition, particularly by the right hemisphere, is an important element in both the execution and simulation of stone tool-making strategies [13, 21]. This might potentially relate to increased functional connectivity with rSFG under some conditions (e.g. for Oldowan strategy vs. prediction following acquisition of flake production skill).

## General Discussion

We have shown that making technical judgments about Lower Paleolithic toolmaking affects neural activity and functional connectivity in dorsal prefrontal cortex, that effect magnitude correlates with the frequency of correct strategic judgments, and that the ability to make such judgments is predictive of success in Acheulean, but not Oldowan, toolmaking. This corroborates hypothesized cognitive control demands of Acheulean toolmaking, specifically including information monitoring and manipulation functions attributed to the "central executive" of working memory.

Stone toolmaking is a demanding technical skill that can take years to master. With an average of 167 hours practice over 22 months, our subjects gained competence in flake production but showed less improvement in handaxe-making. This provides a reference point for estimating the learning investments of Paleolithic toolmakers [17, 34]. Ability to judge strategic appropriateness increased steadily with training, whereas concrete fracture prediction did not, corroborating evidence that technological concepts are more easily acquired than the perceptual-motor skills needed for controlled and predictable flake detachment [17]. Interestingly, training effects in visual cortex (IPS0, MOG) were predictive of success on the Strategy task, suggesting that the education of attention is also an important aspect of such “conceptual” understanding in stone toolmaking. The demands of perceptual-motor skill acquisition should be taken into account when evaluating the cognitive implications of prehistoric technologies, and particularly the self-regulatory capacities and social scaffolding that may have been necessary for sustained, deliberate practice [4, 17].

We did not find a strong relationship between the predictive and strategic abilities measured by our experimental tasks and actual success at Oldowan flaking. This reinforces the point that flake production is a simple technology [64] with limited contingency between successive actions [65, 66], so that unexpected outcomes and sub-optimal choices are easily accommodated if basic requirements for forceful, accurate percussion are met [21]. In contrast, success making technological judgments was consistently predictive of success at handaxe-making. This reflects the fact that bifacial thinning is a more difficult technique requiring the reliable production of particular flake features and contingent action sequences [66], and is consistent with previous imaging studies showing increased prefrontal responses to Acheulean toolmaking [21, 22]. We conclude that explicit prediction and evaluation of toolmaking action outcomes may be unnecessary for effective Oldowan flaking but is a normal part of Acheulean handaxe-making skill.

The three way interaction in lSFG confirms our prediction that the cognitive control demands of toolmaking are modulated by a combination of task, training, and technology. Executive function, including the “central executive” of working memory, is most classically associated with mid-DLPFC but a broader network of regions is clearly relevant [26, 52, 67]. Lesion evidence indicates that lSFG is an important part of this network, specifically contributing to information monitoring and manipulation [51]. We did not anticipate the complexity of the interaction of Time, Task and Technology in lSFG, the details of which may reflect the exploration and even misunderstanding [28] typical of early/intermediate stage learning. Consistent with this interpretation, the relationship of lSFG activity with actual task success rates did meet expectations, being uncorrelated for Prediction but positively correlated for Strategy. This lSFG contribution to strategic evaluation likely arises from the region’s position as a key node for interaction between default and control networks during internally focused, goal-directed cognition [50, 54], and particularly the planning/simulation of future actions [55] (cf. “mental time travel” [68]). Indeed, we found that the lSFG cluster identified in our study had its most salient anatomical connections of with elements of the control and default networks, and modulated its functional connectivity with these networks in response to our experimental tasks and training.

Independent of training, the Strategy task elicited greater functional connectivity with left mid-DLPFC (control network) and medial FPC (default network). Both regions are involved in planning and decision making, with mid-DLPFC classically contributing to monitoring task-relevant information in working memory and FPC to metacognitive management of abstract relations and competing goals [25]. Medial FPC specifically is involved in the manipulation of information held in memory [69], including the prospective memory of planned actions and intentions [70]. In contrast, the Prediction task produced greater functional connectivity with anterior premotor cortex, a region associated with lower-level cognitive control functions such as domain-specific working memory maintenance and action selection based on contextual cues [25]. This dichotomy confirms the task-sensitive coupling of default and control networks via lSFG in our experiment, and supports the hypothesized involvement of abstract information monitoring and manipulation during strategic judgments about Paleolithic toolmaking action plans. Such integration was also evident in the interaction between Task and Time, which involved a subset of default and control regions recently shown to experience coupling during future planning by “process simulation” [55]. The pattern of this interaction suggests that training led subjects to rely less on such prospective simulation when anticipating physical outcomes, but that it continued to be relevant for strategic evaluation.

## Conclusion

Sixty years ago, it was uncontroversial to assert that “Even the crudest Paleolithic artifacts indicate considerable forethought…Using a hammerstone to make a hand-axe, and striking a stone flake to use in shaping a wooden spear, are activities which epitomize the mental characteristics of man” [6: 15]. Although progress in archaeological and comparative research has fostered healthy skepticism regarding such naive appraisals [64], results presented here lend support to the intuitions of an earlier generation and offer hope for further insights into human cognitive evolution. It has been proposed that modern human cognition emerged through changes in prefrontal executive function [1, 3] but that, unfortunately, most behaviors preserved in the archaeological record do not document these changes. Stone tools in particular are seen as products of mundane, over-learnt routines that would not have required flexible cognitive control [1]. This contrasts with the introspection of some toolmakers, who assert that toolmaking “based on raw material which is never standard, and with gestures of percussion that are never perfectly delivered” [27: 117] cannot be reduced to formulaic routines and necessarily involves flexible prospection and planning. We hypothesized that such demands, if present, would have been most pronounced during learning, with effortful cognitive control processes acting as a “scaffold” during unskilled performance [71]. This was confirmed by our results, which show that novice toolmakers rely on the executive functions of lSFG, and particularly its connectivity with functional networks involved in prospective simulation, to make correct strategic judgments. Furthermore, we found that the ability to make such judgments was predictive of success in handaxe-making but not simple flake production. This is consistent with previous findings of greater prefrontal responses to naturalistic Achuelean vs. Oldowan toolmaking, and indicates that the increased cognitive control demands of Acheulean toolmaking specifically include dorsal PFC information monitoring and manipulation. Apart from these specific conclusions, our results more broadly show that it is possible to measure the differential cognitive control demands of even the simplest Lower Paleolithic technologies. This information will not resolve the directionality of causation between technological, cognitive and neuroantomical changes over human evolution, which must be addressed in other ways [7, 23]. What it does is allow for objective comparison of the cognitive control demands of archaeologically observable behaviors, thus expanding the scope of hypotheses regarding the context and timing of evolutionary developments [10] that can be tested using the millions of stone artifacts which dominate the "Stone Age" archaeological record.
