# Correction: Cognitive Demands of Lower Paleolithic Toolmaking

**DOI:** 10.1371/journal.pone.0128256

**Published:** 2015-05-08

**Authors:** The PLOS ONE Staff

## Notice of Republication

This article was republished on April 27, 2015, to correct figures that were distorted during the production process. The publisher apologizes for the error. Please download this article again to view the correct version. The originally published, uncorrected article and the republished, corrected article are provided here for reference.

## Supporting Information

S1 File Originally published, uncorrected article. (PDF)

S2 File Republished, corrected article. (PDF)

## References

[1] Stout D, Hecht E, Khreisheh N, Bradley B, Chaminade T (2015) Cognitive Demands of Lower Paleolithic Toolmaking. PLoS ONE 10(4): e0121804 doi:10.1371/journal.pone.0121804 25875283 10.1371/journal.pone.0121804 PMC4398452

